# Optimizing Response Rates to Examine Health IT Maturity and Nurse Practitioner Care Environments in US Nursing Homes: Mixed Mode Survey Recruitment Protocol

**DOI:** 10.2196/56170

**Published:** 2024-08-29

**Authors:** Gregory L Alexander, Lusine Poghosyan, Yihong Zhao, Mollie Hobensack, Sergey Kisselev, Allison A Norful, John McHugh, Keely Wise, M Brooke Schrimpf, Ann Kolanowski, Tamanna Bhatia, Sabrina Tasnova

**Affiliations:** 1 School of Nursing Columbia University New York, NY United States; 2 Icahn School of Medicine Mount Sinai New York, NY United States; 3 School of Public Health Columbia University Mailman New York, NY United States; 4 Pennsylvania State University University Park, PA United States

**Keywords:** surveys and questionnaires, survey methods, health care surveys, survey, survey design, mixed-mMode survey, nursing homes, nursing home, clinical informatics research, electronic health records, electronic health record, clinicians, HIT Maturity, Care Environments, United States

## Abstract

**Background:**

Survey-driven research is a reliable method for large-scale data collection. Investigators incorporating mixed-mode survey designs report benefits for survey research including greater engagement, improved survey access, and higher response rate. Mix-mode survey designs combine 2 or more modes for data collection including web, phone, face-to-face, and mail. Types of mixed-mode survey designs include simultaneous (ie, concurrent), sequential, delayed concurrent, and adaptive. This paper describes a research protocol using mixed-mode survey designs to explore health IT (HIT) maturity and care environments reported by administrators and nurse practitioners (NPs), respectively, in US nursing homes (NHs).

**Objective:**

The aim of this study is to describe a research protocol using mixed-mode survey designs in research using 2 survey tools to explore HIT maturity and NP care environments in US NHs.

**Methods:**

We are conducting a national survey of 1400 NH administrators and NPs. Two data sets (ie, Care Compare and IQVIA) were used to identify eligible facilities at random. The protocol incorporates 2 surveys to explore how HIT maturity (survey 1 collected by administrators) impacts care environments where NPs work (survey 2 collected by NPs). Higher HIT maturity collected by administrators indicates greater IT capabilities, use, and integration in resident care, clinical support, and administrative activities. The NP care environment survey measures relationships, independent practice, resource availability, and visibility. The research team conducted 3 iterative focus groups, including 14 clinicians (NP and NH experts) and recruiters from 2 national survey teams experienced with these populations to achieve consensus on which mixed-mode designs to use. During focus groups we identified the pros and cons of using mixed-mode designs in these settings. We determined that 2 mixed-mode designs with regular follow-up calls (Delayed Concurrent Mode and Sequential Mode) is effective for recruiting NH administrators while a concurrent mixed-mode design is best to recruit NPs.

**Results:**

Participant recruitment for the project began in June 2023. As of April 22, 2024, a total of 98 HIT maturity surveys and 81 NP surveys have been returned. Recruitment of NH administrators and NPs is anticipated through July 2025. About 71% of the HIT maturity surveys have been submitted using the electronic link and 23% were submitted after a QR code was sent to the administrator. Approximately 95% of the NP surveys were returned with electronic survey links.

**Conclusions:**

Pros of mixed-mode designs for NH research identified by the team were that delayed concurrent, concurrent, and sequential mixed-mode methods of delivering surveys to potential participants save on recruitment time compared to single mode delivery methods. One disadvantage of single-mode strategies is decreased versatility and adaptability to different organizational capabilities (eg, access to email and firewalls), which could reduce response rates.

**International Registered Report Identifier (IRRID):**

DERR1-10.2196/56170

## Introduction

### Background

Survey use in clinical informatics research is ubiquitous. Surveys are often used to collect data and measure phenomena such as knowledge of clinical informatics specialties [1] or the use of electronic health records [2]. Benefits of using surveys include lower costs to conduct research, better population descriptions, flexibility, and dependability of study designs [3]. Surveys are used in many professions and across health care settings, including nursing homes, home health care, and hospitals [4-6]. The expansive use of surveys in clinical informatics research calls for a continued focus on training to improve the ability of researchers to design high-quality surveys, develop effective reporting mechanisms, maximize recruitment strategies, and adapt to recruitment challenges needed to enhance the results. Various modes of survey data collection exist across studies. Literature establishing a theoretical foundation for questionnaire response styles used in surveys when collecting data about public opinion indicate that mode of data collection (eg, mixed-modes) is an important stimulus for response [7]. In this paper, researchers describe a research protocol using mixed-mode survey designs in clinical informatics research using 2 survey tools to explore Health IT (HIT) maturity and nurse practitioner (NP) care environments in US nursing homes (NHs).

In this protocol, HIT maturity is defined in 3 dimensions including HIT capabilities, use, and integration. These HIT maturity dimensions are conceived within NH resident care, clinical support (eg, HIT use in laboratory, pharmacy, and radiology activities), and administrative activities [8]. The HIT maturity survey tool contains 27 content areas and 183 content items [9]. The tool will be used to survey NH administrators. The Nurse Practitioner Nursing Home Organizational Climate Questionnaire (NP-NHOCQ), used to measure NP care environments, contains 5 subscales and 41 items. This tool will be used to survey NPs in NHs. The NP-NHOCQ measures the care environment of NPs in NHs in 5 areas: (1) NP-Physician Relations, (2) NP-Administration Relations, (3) NP-Director of Nursing Relations, (4) Independent Practice and Support, and (5) Professional Visibility.

### Mixed-Mode Survey Research

Survey-driven research is known as a reliable data collection method to capture individual perspectives on a large scale. However, there are many challenges related to survey-based data collection, such as low response rates and rising costs of human capital [10]. Previously, researchers have explored the use of mixed-mode survey designs combining methods such as web, phone, face-to-face, and mail administrations. Mixed-mode survey research involves using 2 or more of these modes for data collection [11]. A survey mode is defined as the communication channel used to collect survey data from one or more respondents [11]. Prior research has reported the benefits of mixed-mode surveys such as enhancing engagement [12], mitigating accessibility barriers [13], and increasing response rates [14].

**Figure 1:**
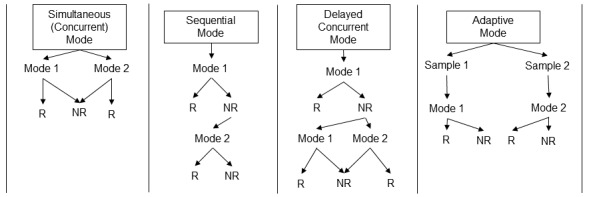
Mixed-mode design strategies (adapted from Schouten et al 2022 [11,15] with permission from Taylor & Francis Group LLC). R: Response; NR: Nonresponse.

Survey modes can be implemented individually or combined with other modes. A single mode approach deploys only one mode at a time. For example, a researcher may use postal mail services as the only method to contact study participants and collect data. Alternatively, mixed-mode designs use multiple modes to recruit respondents (see Figure 1). For instance, a simultaneous (also known as concurrent) mixed-mode approach allows respondents to choose their preference between multiple modes deployed at the same time. For instance, a researcher may offer study participants a choice to complete a survey using an electronic PDF version of the questionnaire that can be printed, scanned, and faxed back to researchers or an electronic survey link completed via web. Mixed-modes can also use a sequential approach. In this mode, researchers may offer 2 different modes, one mode at a time, with a second mode coming later, after the first. This mode is particularly useful when following up with participants who do not respond (nonrespondents) to provide alternative survey strategies that better suit their workflows. An example of sequential mode may include contacting participants initially via phone call and then, following no initial response, a second contact is made using a QR code that is sent via a mailed letter. Another mixed-mode useful for following up with nonrespondents is called a delayed concurrent mode. In this mode, participants are offered one mode, then nonrespondents are offered a choice between 2 other modes later during follow-up activities. An example of the delayed concurrent mode might include an initial mailed survey. Then when no response is received, potential participants are sent a choice between a face-to-face or a phone interview to complete the survey. Finally, an adaptive mixed-mode design incorporates different sampling units. In the adaptive modes, 2 different samples are each offered a different mode.

Mixed-mode survey research has long been identified as a means to improve participation in survey recruitment. For instance, a systematic review of 22 articles among nurses provided evidence that recruitment design strategies that include postal and telephone contacts are generally more successful than fax or web-based approaches [16]. In a more recent systematic review of 893 studies, mode of administration was a key factor in successful recruitment. However, in this review, electronic and postal modes of survey data collection were less likely to result in higher response rates [17]. In other research using mixed-modes with clinicians, using a multiple contact protocol generated final response rates 10% points higher than single mode methods [18].

In this paper, we present mixed-mode methods used in a large survey of NHs in the United States. To achieve research goals, we must have a robust and effective recruitment plan. Therefore, we are using an innovative research protocol using mixed-modes to improve NH administrators’ and NPs’ engagement in survey data collection while increasing the response rates.

## Methods

### Mixed-Mode Survey Research in Nursing Homes

The US health care system has over 15,600 NHs serving over 1.3 million residents [19]. A growing strategy for improving the outcomes for NH residents is to effectively integrate HIT into care delivery to promote safer care environments for NH residents. HIT integration into NH resident care may improve care environments and by extension, better care quality [20]. Survey-driven research is a reliable method to capture the perspective of individuals about these phenomena on a large scale. Our team is conducting a national survey of NH administrators and NPs, incorporating 2 different survey tools to explore how HIT maturity (survey 1) impacts care environments (survey 2) where NPs work. A specific aim of this research is to provide comprehensive assessments of HIT maturity and NP care environments in NHs nationally. The goal of the National Institute of Aging funded research study (5R01AG080517, principal investigators: GLA and LP) is to assess differences in HIT maturity and care environments in NHs where NPs deliver care to residents with Alzheimer disease and related dementias and examine their impact on hospitalizations and emergency department visits among residents.

### Sample

#### Overview

The sample for this study includes randomly selected NHs including administrators (ie, NH leaders responsible for HIT systems in their organization) and NPs from each NH. Our goal is to recruit participants from 1400 NHs in the United States. We use 2 national sources to identify NHs for this study. The first data source is called NH Compare (or Care Compare), a publicly available national data set containing information about organizational characteristics of US NHs and quality of care [21]. The second data source stems from IQVIA, a company that stores national data about NH location, contact information, and staff including administrators and NPs. In preparation for this proposal, IQVIA provided our team data to identify all US NHs with practicing NPs. According to these data, in 2021, a total of 11,222 unique NPs worked in 5000 NHs for an average of 2.2 NPs/NH. Based on this estimate, we expect to contact 3080 NPs within the 1400 NHs (1400 NHs X 2.2 NPs/NH).

#### Inclusion and Exclusion Criteria

We use NH Compare files to identify NHs for our study based on 2 specific inclusion criteria. First, we include all NHs located in the United States including Alaska and Hawaii. Second, we include at least 1 NP working in each facility. NPs may include actual employees of a facility or may be employed by an external organization as a consultant for a facility and not directly by the NH. Facilities are not eligible to participate if they meet the following 3 exclusion criteria. First, NHs that do not have an NP employed. Second, NHs with a hospital-based designation as their HIT maturity are likely to be different due to national incentives for HIT adoption in acute care [22,23]. Approximately 6% (n=15,518) of NHs have a health system designation that includes common ownership or joint management [24]. Third, NHs that are designated as a special focus facility (SFF), which indicates any NH with a history of serious quality issues. NHs with an SFF designation are required to be in a program to stimulate quality-of-care improvements [25]. In October 2023, Centers for Medicare & Medicaid Services indicated that approximately 0.5% of US NHs have an SFF designation [25].

The NH Compare website was downloaded in February 2023 to identify facilities for recruitment. We identified 4163 facilities that matched our criteria. In preliminary work, during 2 prior NH survey studies, we achieved approximately a 45% response rate of surveys returned from administrators. Therefore, for the current protocol, we oversampled by randomly selecting 3000 NHs, which we identified by linking the NH Compare and IQVIA data. We included at least 5 facilities in each state, except for Alaska (2 facilities) and Wyoming (3 facilities) which have few NHs with NPs identified. We will recruit all administrators from these 3000 NHs to complete a HIT maturity survey. For every NH that completes the HIT maturity survey, we will recruit all NPs from those facilities.

#### Sample Characteristics

After we generated the random sample from the merged files, we compared basic characteristics of NHs between the selected NHs and the rest of the NHs nationally. The following NH characteristics were compared to assure that there was limited bias in sample representation:

1. Bed size (<60 beds, 60-120 beds, and >120 beds)
2. Ownership (for profit vs nonprofit)
3. Location (metropolitan, micropolitan, small town, and rural)
4. Staffing hour
5. Medicare vs Medicaid
6. NH overall rating: (ranging from 1 to 5)

### Focus Groups to Assess the Pros and Cons of Mixed-Mode Designs

The research team conducted iterative focus groups that included NPs and survey recruitment experts to discuss the pros and cons of different recruitment strategies. To explore the pros and cons, members of the focus groups assessed recruitment strategies used during 2 prior national studies of long-term care NH sites [26]. The PI and some members of the focus groups lead these national studies that were reviewed. Additionally, members of the focus groups reviewed and discussed potential mixed-mode strategies from the literature to incorporate into this protocol. Schouten [15] mixed-mode survey research helped inform our decisions for our protocol design.

### Data Collection

**Figure 2:**
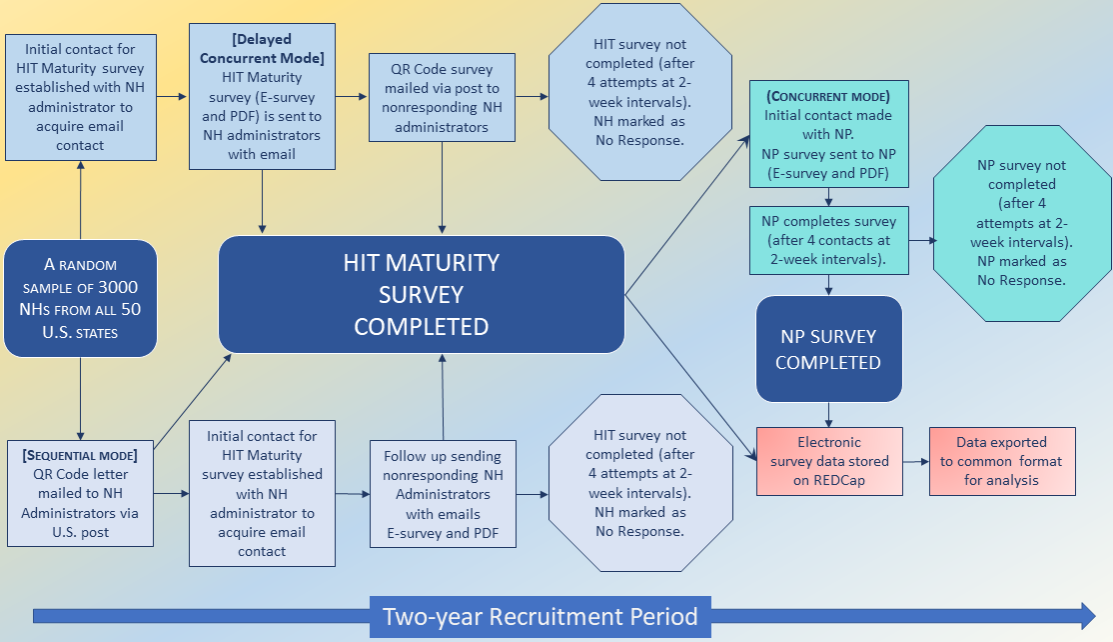
Survey recruitment protocol for hybrid mixed-mode design and data collection workflow NH: Nursing Home, NP: Nurse Practitioner, E-survey: Electronic Survey, QR Code: QR Code Letter Mailed, HIT: Health IT.

We aim to survey administrators and NPs using 2 survey tools describing HIT maturity and care environments from each discipline, respectively. To prepare the protocol, the research team conducted 3 iterative focus groups with clinicians (NPs and NH experts), recruiters from 2 national survey teams experienced with recruitment in NHs and with NPs, and a statistician to achieve consensus on which mixed-mode designs to incorporate into this research. Our research protocol workflow is illustrated in Figure 2. The following sections include descriptions of the mixed-mode workflows by discipline and the surveys being used in this protocol.

### Survey 1: NH Administrator and HIT Maturity

For each randomly selected NH, contact information for NH Administrators has been obtained using IQVIA data set. Our team searched NH websites to confirm contact information of current administrators. During initial contact with each NH administrator (either by phone or a mailed letter), we describe the study’s purpose and explain the study. All administrators who are contacted and agreed to participate in the study will be sent a cover letter providing details about the study’s purpose, instructions on how to complete the NH HIT maturity survey tool, and descriptions of the benefits and risks of participation. We provide a description of the HIT maturity survey for administrators including that the survey measures HIT capabilities, extent of HIT use, and degree of HIT integration in resident care, clinical support, and administrative activities [9]. We incorporate 2 mixed-mode designs when recruiting NH administrators including a Delayed Concurrent Mode and a Sequential Mode with regular follow-up phone contacts to stimulate engagement.

#### Delayed Concurrent Mode

Our primary mode for this study is a Delayed Concurrent mixed-mode design. In this mode, administrators are offered the choice between multiple modes. During the first contact (conducted by phone), we describe the project and obtain email addresses for administrators who agree to participate. Then, we follow up with administrators by email with an electronic survey link and a PDF simultaneously. This is important because the different choices among electronic surveys and PDFs allow administrators the flexibility to choose a mode that fits their needs. In nonresponse cases, administrators are later offered a different mode including a postal letter with a QR code that includes a URL link to the survey tool that is subsequently sent at a later time.

#### Sequential Mode

As a secondary option, we incorporate a Sequential Mode for a minimum of 10% of the facilities in each state. In this mode, participants are offered only 1 mode at a time and only part of the nonrespondents are invited for the second mode. The first mode includes mailing a postal letter that describes the study and provides both a QR code and URL link to the survey for the NH administrator. Recruiters make a series of follow-up calls to administrators after the letter is sent. During follow-up calls, emails are confirmed by the recruitment team. In this sequence, administrators who agree to take the survey and have provided their email addresses but have failed to respond with a completed mailed or faxed survey after a minimum of four follow-up calls are offered a second mode. The second mode includes a URL link and a PDF of the survey that is sent to administrators via email.

### Survey 2: NPs and Care Environment

#### Overview

The recruitment team asks administrators to confirm that at least one NP works in their facility (whether employed by the NH or by an external health organization that provides NP services to the facility) and to verify the NP’s name and contact information. NP’s contact information listed in the IQVIA data is confirmed with administrators to ensure that it is current. Contact information that is not current is updated by the recruitment team in the recruitment database. NHs that do not meet the eligibility criterion (eg, NP left and no new NP hired) are excluded. The research team will incorporate a concurrent mixed-mode design to recruit NPs for the study.

#### Concurrent Mode

NPs are contacted by email or phone by our recruitment team and are provided with information describing the study, its voluntary nature, and confidentiality per the institutional review board’s (IRB’s) protocol. NPs are sent links to both an electronic survey and PDF concurrently. We expect some NHs to have more than one NP complete a survey.

### Ethical Considerations

The protocol was approved by the IRB (AAAU3845). Ethical issues that were addressed in our IRB protocol included confidentiality and anonymity of privacy to encourage honest responses. Security and accessibility of the data only to authorized research staff. Researchers also created plans for minimizing coercive behaviors during recruitment (eg, applying pressure) by creating systematic follow-up and templates with recruitment language to use during contacts. The research protocol and all procedures were approved by Columbia University IRB (AAAU3845).

### Follow-Up and Engagement

Up to 4 follow-up phone calls are conducted at specified 2-week intervals for administrators who have agreed to participate. Administrators and NPs who do not complete surveys are marked as “No Contact.” Administrators and NPs who complete a survey receive US $25 compensation in the form of a gift card.

### Survey Coding and Cleaning

All survey data collection is conducted through REDCap (Research Electronic Data Capture; Vanderbilt University) a web-based software designed for data collection and management in research studies with emphasis on data security and flexibility [27]. We maintain data about recruitment efforts in REDCap, including number of facilities contacted, persons contacted at each facility, packets or links sent, surveys received, initial cannot reach, contact calls made, follow-up calls made, confirmations received (will complete and not completed), stated completions, and follow-up cannot reach. Recruitment staff, including a project coordinator and 4 research assistants, make recruitment calls and send surveys to NH administrators and NPs.

Data collected via electronic survey are electronically transferred to the REDCap database. Data collected via PDF is manually entered into the REDCap system by our research staff. A meticulous data-cleaning strategy is used before formal statistical analysis to ensure the data quality [28]. We used algorithms to check questionnaires for consistency and validity. For example, graphical exploration through boxplots, histograms, and scatter plots will be used to help with detecting outliers and logically implausible data points. Any identified outlying observations undergo thorough examination to discern between potential data entry errors and genuinely extreme values. Data entry errors are corrected. Any systematic patterns will be scrutinized. Every step of the data cleaning process and associated decisions are documented to ensure transparency.

There is a possibility that some NH administrators or NPs who agree to participate in the study will not fill out an HIT maturity or care environment survey tool completely. We anticipate that there may be some missing data on completed surveys. Based on prior national HIT maturity and NP studies, we have estimated that less than 3% of the data for surveys received was missing for both types of surveys. We plan to use all available data in our analyses.

### Survey Measures

NH HIT Maturity [8,29] is measured using a total composite score that correspond to 7 HIT maturity stages. The 7 maturity stages range from the lowest HIT Maturity Stage 0—Nonexistent HIT solutions or electronic health records to Stage 6—Use of data by residents and resident representatives to generate clinical data and drive self-management. A higher total HIT maturity score indicates greater IT capabilities, use, and integration in resident care, clinical support (including IT systems in pharmacy, radiology, laboratory), and administrative activities in the NH. The overall standardized Cronbach α for this instrument in past research was 0.86 (high); each dimension or domain achieved a Cronbach α ranging from 0.7 to 0.9 [30].

NP Care Environment is measured by a 44-item Nurse Practitioner Nursing Home Organizational Climate Survey (NP-NHOCS) [31], which asks NPs to rate the work attributes in NHs using a 5-point Likert scale. The NP-NHOCS has five subscales: (1) NP-Physician Relations (7 items)—measures the relationship, communication, and teamwork between NPs and physicians; (2) NP-Administration Relations (11 items)—measures collaboration and communication between NPs and managers; (3) NP-Director of Nursing Relations (8 items)—measures the relationship, communication, and teamwork between NPs and Directors of Nursing; (4) Independent Practice and Support (9 items)—measures resources and support NPs have for their independent practice; and (5) Professional Visibility (9 items)—measures how visible the NP role is in the organization. We first compute NP-level and then NH-level mean scores by aggregating the responses of all NPs in the NHs as recommended [32]. Higher mean scores indicate better care environments. NPs are asked to complete measures of demographics (eg, age, sex, and experience).

### Analysis

A number of planned analyses will be performed. In terms of HIT maturity survey, we aim to understand which survey mode (Delayed Concurrent vs Sequential) will maximize NHs’ engagement in our research project and which factor(s) influence survey completion method. First, descriptive statistics will be used to summarize the key variables of interest including but not limited to response rates (agreeing to participate or not), completion rates, time taken to complete the survey, and the proportion of electronic surveys received. Chi-square test or Fisher exact test will be used to examine differences in response rates, completion rates, proportion of electronic survey received between the NHs assigned to the Delayed Concurrent Mode and Sequential Mode survey designs. This analysis will determine if one survey mode yields higher response and completion rates compared to the other. Second, if there is sufficient data availability, linear regression models will be used to test whether NH administrators’ demographic characteristics (ie, age, sex, race or ethnicity), NH-level characteristics (eg, bed size and staffing hours), and HIT maturity level are associated with the choice of survey completion method (electronic or PDF format).

In terms of NP care environment survey, all NPs will be offered both an electronic survey and a PDF concurrently. The proportion of electronic surveys received among those who respond will be calculated to determine preference for electronic over PDF surveys. If a sufficient number of electronic and PDF surveys are received, linear mixed effects models with NH as random effect will be used to assess whether the choice of survey completion method is associated with NH-level characteristics (eg, HIT maturity score, geographical location, ownership), NP-level characteristics (eg, age, race or ethnicity, years of experience, and job roles), and NP care environment scores, respectively.

## Results

The research team conducted 3 iterative focus groups with a total of 14 clinicians including NPs and survey recruitment experts. The following pros and cons were used to determine our recruitment strategies.

### Pros of Mixed-Mode Designs

The pros of mixed-mode designs identified by the team during focus groups were that delayed concurrent, concurrent, and sequential mixed-mode approaches can save recruitment time compared to single mode delivery methods. Additionally, effort on the part of recruitment staff is minimized by using mixed-modes. By using mixed survey modes, participants can immediately choose their preferred survey method, potentially enhancing their satisfaction with the survey process. This facilitates engagement that leads to completed surveys and increased response rates. Another pro of the concurrent mode identified was that sending a QR code via the postal service in addition to providing a URL link provides greater selectivity and plasticity in a respondent’s choice, which could enhance engagement and responsiveness to surveys. A pro of single mode designs is the potential for quick turnaround times and representative samples for projects with limited resources [33].

### Cons of Mixed-Mode Designs

One disadvantage of single mode strategies is that they decrease the versatility and adaptability to different organizational capabilities (eg, access to email and system firewalls), which could reduce response rates. For example, a URL link sent via email might be more difficult for NH administrators and NPs to open due to system firewalls put into place by organizations to meet higher level security standards of HIT systems. We identified another con of a sequential mode; for instance, if a singular mode is offered when initial recruitment is started, the respondent may not engage in the second wave. For example, if respondents are concerned about access to email, they may not engage with us again in further calls if the first mode offered including email is perceived as a barrier to participation. Other cons that were identified related to NH infrastructure and environmental variables. For instance, NPs might have limited access or no workspace available to print a PDF and to complete a survey. Other reported cons of mixed-mode designs (sequential modes [web then telephone]) compared to single mode (telephone only) include higher missing data rates and more focal responses [34].

After randomization, we rigorously compared selected and nonselected NHs based on key NH level characteristics such as bed size, ownership, location, staffing hours, payer mix, and overall rating. Our analysis did not reveal statistically significant differences in these characteristics (See Table S1 in Multimedia Appendix 1).

The research study was funded in February 2023. Participant recruitment for the project began in June 2023. As of June 3, 2024, a total of 109 HIT maturity surveys and 83 NP surveys have been returned. About 69% of the HIT maturity surveys have been submitted using the electronic link and 27% were submitted after a QR code was sent to the administrator. About 95% of the NP surveys were returned with electronic survey links.

## Discussion

Our national study is the first to our knowledge to focus on NH HIT maturity and NP care environments where administrators and NPs work. Although NPs are a predominant provider in NHs [35], no study to date has focused on NP care environments and available resources (eg, technology) to this discipline, leading to limited understanding of how NPs conduct work, and how HIT maturity contributes to an NP’s ability to improve care and outcomes for NH residents with serious chronic conditions. Furthermore, a primary objective of this study is to provide evidence of how administrators and NPs codesign technologies that can transform care delivery in NHs. Our team anticipates that using mixed-modes will enhance our ability to work with participants at different stages of HIT maturity, which we believe is in an important factor in how care environments are perceived by employees (eg, NPs) in these settings.

To achieve this goal, we first must be able to maximize engagement in this survey research with strong representation by both NH administrators and NPs from all US states. Second, we must mitigate barriers to NH administrators and NPs accessing surveys so that they can participate. Finally, we must achieve acceptable response rates by generating different modes of support, providing choice and flexible means for NH administrators and NPs to participate in the survey process. In this protocol, we have identified mixed-mode recruitment strategies based on the expert opinion of experienced survey recruitment staff that should enable us to meet our goals and to achieve a representative national sample of NH administrators and NPs.

### Limitations

This study may have limitations. In prior work, we have identified great variability in HIT capabilities among many NH's, such as access to external email and connectivity challenges where NH staff work [36]. Depending on the survey mode used during the data collection, this variation may create differences in response rates between facilities. We have incorporated various mixed-mode methods in this research protocol that should allow respondents to choose their preferred method and the ability to complete a survey considering their institutional characteristics. The use of mixed-modes has been shown to improve participation in survey research, thus reducing barriers for less well-resourced NHs (eg, NHs with lower HIT maturity levels). Less resourced NHs are typically those with greater resident ethnic and racial diversity [37], so improving their participation is critical to enhance representation of these communities, which is a benefit of the design.

### Conclusions

This research protocol describes a study using 2 survey tools to measure HIT maturity and NP care environments in the US NHs as perceived by administrators and NPs. We have identified the pros and cons of survey recruitment strategies experienced by our team in past work. We reviewed evidence-based recruitment strategies using mixed-modes which are defined in the literature as methods that incorporate the use of 2 or more modes to recruit respondents. In this protocol, we have incorporated a delayed concurrent mode, sequential mode, and a concurrent mode to enhance engagement, mitigate barriers to survey access, and to increase response rates in collecting survey data both from NH administrators and NPs to have robust data for future analysis.

## Appendix

Multimedia Appendix 1 Supplementary Table.

## Abbreviations

HIT: health IT
IRB: institutional review board
NH: nursing home
NP: nursing practitioner
NP-NHOCQ: Nurse Practitioner Nursing Home Organizational Climate Questionnaire
NP-NHOCS: Nurse Practitioner Nursing Home Organizational Climate Survey
REDCap: Research Electronic Data Capture
SFF: special focus facility
