# Supplementary material for: Optimizing Response Rates to Examine Health IT Maturity and Nurse Practitioner Care Environments in US Nursing Homes: Mixed Mode Survey Recruitment Protocol
Source: JMIR Res Protoc. 2024 Aug 29;13:e56170. doi: 10.2196/56170 (PMC11393505; doi:10.2196/56170)
Supplement: Multimedia Appendix 1 [file resprot_v13i1e56170_app1.docx]

Table S1: Comparison of Characteristics from Selected and Non-selected Nursing Homes Eligible for the Study

|  | Not Selected (N=1463) | Selected (N=3000) | P-value |
| --- | --- | --- | --- |
| **Occupancy.rate** |  |  |  |
| Mean (SD) | 0.765 (0.154) | 0.767 (0.163) | 0.775 |
| Median [Min, Max] | 0.790 [0.100, 1.20] | 0.790 [0.0297, 2.64] |  |
| Missing | 1 (0.1%) | 9 (0.3%) |  |
| **Total.nursing.staff.turnover** |  |  |  |
| Mean (SD) | 53.4 (15.0) | 53.9 (15.3) | 0.352 |
| Median [Min, Max] | 53.0 [15.7, 100] | 53.0 [8.50, 100] |  |
| Missing | 210 (14.4%) | 429 (14.3%) |  |
| **Registered.Nurse.turnover** |  |  |  |
| Mean (SD) | 52.8 (19.7) | 52.8 (20.4) | 0.996 |
| Median [Min, Max] | 50.0 [0, 100] | 52.2 [0, 100] |  |
| Missing | 248 (17.0%) | 532 (17.7%) |  |
| **Total.Number.of.Penalties** |  |  |  |
| Mean (SD) | 2.25 (2.83) | 2.29 (3.21) | 0.671 |
| Median [Min, Max] | 1.00 [0, 33.0] | 1.00 [0, 50.0] |  |
| **Number.of.Facility.Reported.Incidents** |  |  |  |
| Mean (SD) | 1.44 (2.92) | 1.35 (3.14) | 0.368 |
| Median [Min, Max] | 0 [0, 29.0] | 0 [0, 43.0] |  |
| **Five.Star.Staffing.Rating** |  |  |  |
| 1 | 415 (28.4%) | 879 (29.3%) | 0.454 |
| 2 | 391 (26.7%) | 727 (24.2%) |  |
| 3 | 292 (20.0%) | 593 (19.8%) |  |
| 4 | 241 (16.5%) | 532 (17.7%) |  |
| 5 | 114 (7.8%) | 238 (7.9%) |  |
| Missing | 10 (0.7%) | 31 (1.0%) |  |
| **Ownership** |  |  |  |
| For profit | 1092 (74.6%) | 2252 (75.1%) | 0.786 |
| Non profit | 371 (25.4%) | 748 (24.9%) |  |
| **Bed.size** |  |  |  |
| < 60 | 112 (7.7%) | 254 (8.5%) | 0.647 |
| >120 | 631 (43.1%) | 1278 (42.6%) |  |
| 60-120 | 720 (49.2%) | 1468 (48.9%) |  |
| **Provider.Type** |  |  |  |
| Medicaid | 10 (0.7%) | 34 (1.1%) | 0.345 |
| Medicare | 51 (3.5%) | 99 (3.3%) |  |
| Medicare and Medicaid | 1402 (95.8%) | 2867 (95.6%) |  |
| **Location by Rural Urban Commuting Area (RUCA*) Codes** |  |  |  |
| Metropolitan | 1048 (71.6%) | 2108 (70.3%) | 0.521 |
| Micropolitan | 149 (10.2%) | 308 (10.3%) |  |
| Rural | 50 (3.4%) | 89 (3.0%) |  |
| Small Town | 74 (5.1%) | 179 (6.0%) |  |
| Missing | 142 (9.7%) | 316 (10.5%) |  |
| ****CNA Hours/Resident/Day** |  |  |  |
| high | 465 (31.8%) | 990 (33.0%) | 0.595 |
| low | 482 (32.9%) | 973 (32.4%) |  |
| Mid | 490 (33.5%) | 964 (32.1%) |  |
| Missing | 26 (1.8%) | 73 (2.4%) |  |
| **^$^RN Hours/Resident/Day** |  |  |  |
| high | 10 (0.7%) | 24 (0.8%) | 0.899 |
| low | 1422 (97.2%) | 2892 (96.4%) |  |
| Mid | 5 (0.3%) | 11 (0.4%) |  |
| Missing | 26 (1.8%) | 73 (2.4%) |  |
| **^$^LPN Hours/Resident/Day** |  |  |  |
| high | 476 (32.5%) | 979 (32.6%) | 0.64 |
| low | 469 (32.1%) | 986 (32.9%) |  |
| Mid | 492 (33.6%) | 962 (32.1%) |  |
| Missing | 26 (1.8%) | 73 (2.4%) |  |
| **^$^CNA_adjusted Hours/Resident/Day** |  |  |  |
| high | 467 (31.9%) | 986 (32.9%) | 0.662 |
| low | 490 (33.5%) | 963 (32.1%) |  |
| Mid | 478 (32.7%) | 974 (32.5%) |  |
| Missing | 28 (1.9%) | 77 (2.6%) |  |
| **^$^RN_adjusted Hours/Resident/Day** |  |  |  |
| high | 12 (0.8%) | 29 (1.0%) | 0.744 |
| low | 1414 (96.7%) | 2871 (95.7%) |  |
| Mid | 9 (0.6%) | 23 (0.8%) |  |
| Missing | 28 (1.9%) | 77 (2.6%) |  |
| **^$^LPN_adjusted Hours/Resident/Day** |  |  |  |
| high | 473 (32.3%) | 980 (32.7%) | 0.413 |
| low | 465 (31.8%) | 988 (32.9%) |  |
| Mid | 497 (34.0%) | 955 (31.8%) |  |
| Missing | 28 (1.9%) | 77 (2.6%) |  |
| **Quality Measure Five Star Rating)** |  |  |  |
| 1 | 71 (4.9%) | 147 (4.9%) | 0.549 |
| 2 | 191 (13.1%) | 415 (13.8%) |  |
| 3 | 296 (20.2%) | 658 (21.9%) |  |
| 4 | 429 (29.3%) | 833 (27.8%) |  |
| 5 | 471 (32.2%) | 930 (31.0%) |  |
| Missing | 5 (0.3%) | 17 (0.6%) |  |
| **Overall Five Star Rating** |  |  |  |
| 1 | 357 (24.4%) | 702 (23.4%) | 0.454 |
| 2 | 299 (20.4%) | 680 (22.7%) |  |
| 3 | 310 (21.2%) | 632 (21.1%) |  |
| 4 | 253 (17.3%) | 480 (16.0%) |  |
| 5 | 242 (16.5%) | 500 (16.7%) |  |
| Missing | 2 (0.1%) | 6 (0.2%) |  |
| **Long.Stay.Quality Measure Five Star Rating** |  |  |  |
| 1 | 113 (7.7%) | 255 (8.5%) | 0.361 |
| 2 | 205 (14.0%) | 433 (14.4%) |  |
| 3 | 246 (16.8%) | 552 (18.4%) |  |
| 4 | 361 (24.7%) | 678 (22.6%) |  |
| 5 | 510 (34.9%) | 1014 (33.8%) |  |
| Missing | 28 (1.9%) | 68 (2.3%) |  |
| **Short.Stay.Quality Measure Five Star Rating** |  |  |  |
| 1 | 99 (6.8%) | 193 (6.4%) | 0.75 |
| 2 | 167 (11.4%) | 362 (12.1%) |  |
| 3 | 290 (19.8%) | 621 (20.7%) |  |
| 4 | 318 (21.7%) | 622 (20.7%) |  |
| 5 | 464 (31.7%) | 905 (30.2%) |  |
| Missing | 125 (8.5%) | 297 (9.9%) |  |

**Table Key:**

*RUCA1_Recode: Rural-Urban Commuting Area Definition:

RUCA1_recode categorizes areas based on Rural-Urban Commuting Area (RUCA) definitions. Specifically, metropolitan area is defined as area with a primary flow of at least 10% to an urbanized area. Micropolitan area is defined as area with a primary flow of at least 10% to a large urban cluster of 10,000 to 49,999 people. Small town is defined as area with primary flow of at least 10% to a small urban cluster of 2,500 to 9,999 people. Rural regions are the areas with primary commuting flow to a tract outside an urban area or urban cluster.

**"RN_reported", “LPN_reported” and “CNA_reported” variables reported in Staffing Hours per Resident per Day divided into three groups - "low," "mid," or "high" - based on tertiles (lower and upper third boundaries) of the corresponding distributions, respectively.

^$^Similarily, "RN_adjusted", “LPN_adjusted” and “CNA_adjusted” variables Adjusted Staffing Hours per Resident per Day divided into "high," "mid," or "low" groups based on tertiles of the corresponding distributions.
